# From economic survival to recreation: contemporary uses of wild food and medicine in rural Sweden, Ukraine and NW Russia

**DOI:** 10.1186/s13002-015-0036-0

**Published:** 2015-06-16

**Authors:** Nataliya Stryamets, Marine Elbakidze, Melissa Ceuterick, Per Angelstam, Robert Axelsson

**Affiliations:** Swedish University of Agricultural Sciences, Forest-Landscape-Society Research Group, Faculty of Forest Sciences, School for Forest Management, PO Box 43, Skinnskatteberg, SE 73921 Sweden; Research Institute for Nature and Forest, Kliniekstraat 25, Brussels, 1070 Belgium; Nature reserve “Roztochya”, Sitchovuh Strilciv 7, Ivano-Frankove, 81070 Ukraine

## Abstract

**Background:**

There are many ethnobotanical studies on the use of wild plants and mushrooms for food and medicinal treatment in Europe. However, there is a lack of comparative ethnobotanical research on the role of non-wood forest products (NWFPs) as wild food and medicine in local livelihoods in countries with different socio-economic conditions. The aim of this study was to compare the present use of wild food and medicine in three places representing different stages of socio-economic development in Europe. Specifically we explore which plant and fungi species people use for food and medicine in three selected rural regions of Sweden, Ukraine and the Russian Federation.

**Methods:**

We studied the current use of NWFPs for food and medicine in three rural areas that represent a gradient in economic development (as indicated by the World Bank), *i.e.*, Småland high plain (south Sweden), Roztochya (western Ukraine), and Kortkeros (Komi Republic in North West Russia). All areas were characterised by (a) predominating rural residency, (b) high forest coverage, and (c) free access to NWFPs. A total of 205 in-depth semi-structured interviews were conducted with local residents in the three study areas. The collected NWFPs data included (1) the species that are used; (2) the amount harvested, (3) uses and practices (4) changes over time, (5) sources of knowledge regarding the use of NWFPs as wild food and medicine and (6) traditional recipes.

**Results:**

In Sweden 11 species of wild plant and fungi species were used as food, and no plant species were used for medicinal purposes. In Ukraine the present use of NWFPs included 26 wild foods and 60 medicinal species, while in Russia 36 food and 44 medicinal species were reported.

**Conclusions:**

In the economically less developed rural areas of Ukraine and Russia, the use of NWFPs continues to be an important part of livelihoods, both as a source of income and for domestic use as food and medicine. In Sweden the collection of wild food has become mainly a recreational activity and the use of medicinal plants is no longer prevalent among our respondents. This leads us to suggest that the consumption of wild food and medicine is influenced by the socio-economic situation in a country.

## Background

Wild food and medicines are plant and fungal resources that grow in natural conditions and which are harvested or collected for the purpose of human consumption and used as food, dietary supplements and medical treatments [1, 2]. For the purpose of this study animal resources were not included. ‘Wild’ refers to the fact that these species grow without being cultivated [3], however, semi-domesticated species are also included in this definition. The Food and Agriculture Organisation of the United Nations (FAO) proposed the term Non-Wood Forest Products (NWFPs) for goods of biological origin other than wood, derived from forests and other woodlands [4].

NWFPs have contributed to sustaining the livelihoods of people living in forest and woodland landscapes for centuries [5–8]. In Europe, the practice of collecting NWFPs for wild food and medicine has been declining due to economic development leading to urbanization, mass production of food and modern synthetically produced medicines [3, 9, 10]. However, as the health industry has developed over the last few decades, alarms about unhealthy additives in mass produced food have resulted in a renewed interest in wild food and medicine [11, 12]. Wild food is considered pure, naturally healthy and rich in vitamins and antioxidants [13]. Moreover, wild plants and mushrooms play an important role as spices and accompaniments in traditional cuisines in Europe [3, 13–16]. While wild food is becoming a fashionable product [3, 17] in many European countries with a market economy, it is still an important resource for local livelihoods in countries in transition from a planned economy towards a market economy [15, 18–21]. There is also a growing interest in traditional medicine in different parts of Europe [3, 13, 17, 22–25], even where collecting plants for medicinal purposes is no longer a widespread practice [3, 9, 26, 27].

There are many ethnobotanical studies on the use of wild plants and mushrooms for food and medicinal purposes in Europe [6, 15, 16, 19, 24, 28–43]. However, there is a lack of comparative ethnobotanical research on the role of NWFPs as wild food and medicine in local livelihoods in countries with different socio-economic conditions [13, 33, 44, 45].

The aim of this study is to document and analyse current uses of NWFPs as wild food and medicine in three European countries that are in different stages of socio-economic development. We used three rural case studies, located in Sweden, Ukraine and the Russian Federation that represent a gradient in economic development from market economy with intensive industrial management of forests and agricultural land to countries in transition from planned economy to market economy with still existing traditional use of forest and agricultural landscapes [46, 47]. The following research questions will be answered here: Which plant and fungi species do rural people use for food and medicine in the selected rural regions of Sweden, Ukraine and Russia? Do people keep traditional knowledge related to wild wood and medicine? Are there any similarities or differences in the use of wild food and medicine among places with different socio-economic conditions?

## Methods

### Study areas

The Småland high plain (hereafter Småland) in south Sweden, the Roztochya upland region (hereafter Roztochya) in western Ukraine and the Kortkeros rayon (municipality) (hereafter Kortkeros) in the Komi Republic in North West (NW) Russia were selected as case study areas. The selected study areas are all dominated by rural residency, have a high percentage of forest coverage, and free access to forest that provide NWFPs. In all three study areas NWFPs have traditionally been important sources of wild food and medicine for centuries [6, 15, 16, 19, 35, 48–50].

**Fig. 1:**
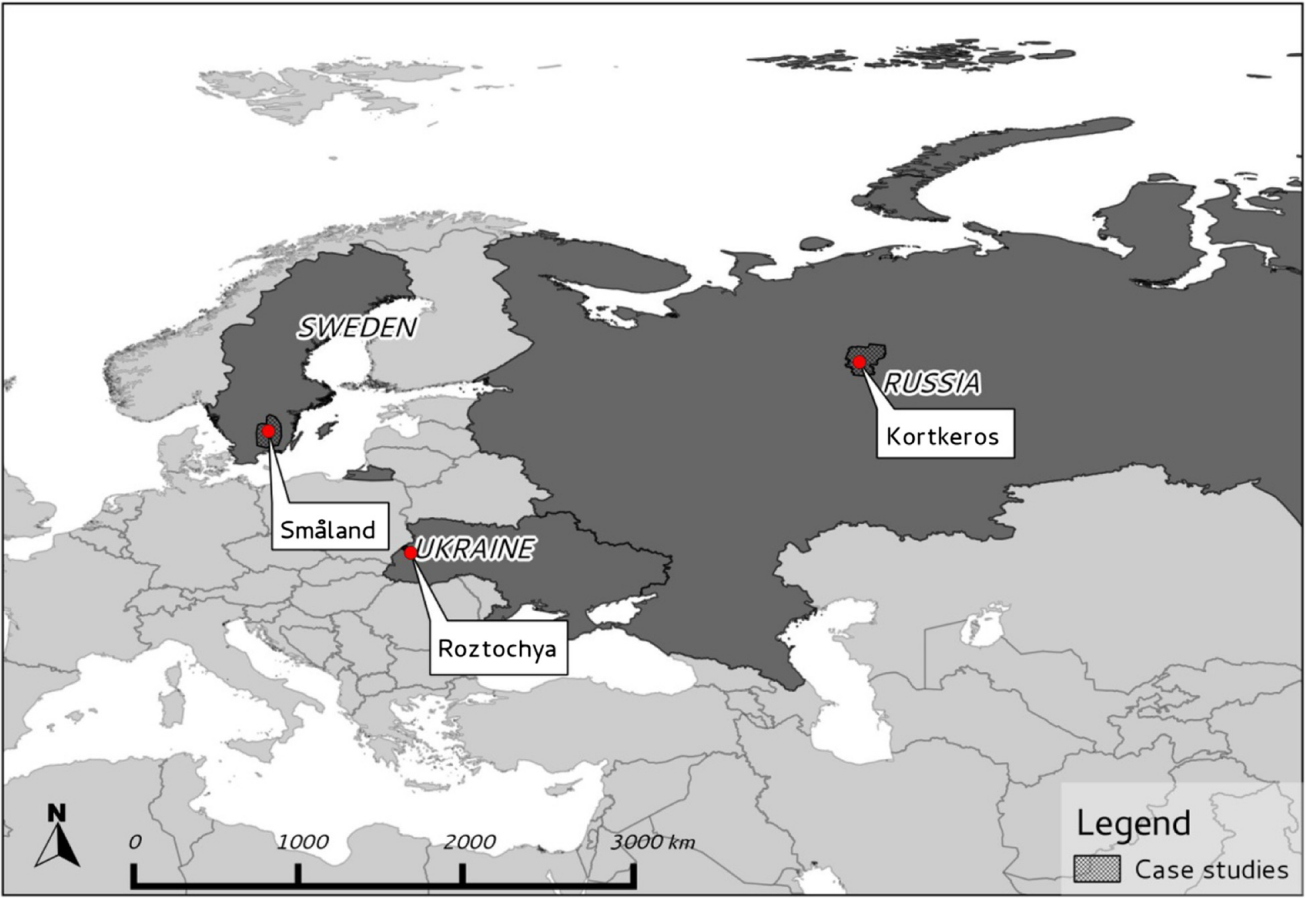
Case studies in Sweden, Ukraine and NW Russia

Småland (56°52′- 57°26′ N and 14°43′-15°04′ E) (Fig. 1) encompasses 1792 km^2^. Boreal forest forms the main land cover, and occupies more than 50 % of the study area. Forests are dominated by Scots pine [*Pinus sylvestris* L.] and Norway spruce [*Picea abies* (L.) Karst.]. The study area is surrounded by hemiboreal forests and temperate lowland deciduous forests with beech [*Fagus silvatica* L.] in the south. In the past this forest-dominated landscapes was used for traditional animal husbandry and farming [51, 52]. Non-industrial private owners own 80–85 % of the forests in the study area [53]. Other main forest owners are the state-owned forest company Sveaskog Co., municipalities and the Swedish Church. This study area includes 22 parishes with an average population density of 53 inhabitants per km^2^, but with only 13 per km^2^ in rural areas [54]. The population trend is negative, especially in rural areas that presently host 26 % of the population in the study area [54]. Nevertheless, unemployment rates are lower than the Swedish average. As everywhere in Sweden, the social security and health care systems are well developed with insurance systems, unemployment funds and support grants available for all people in need [55].

Roztochya (50°06′ N - 49°06′ N and 23°20′ E - 23°54′ E) is situated in the temperate lowland forest ecoregion, and covers 992 km^2^ (Fig. 1). Forests cover about 44 % of the study area. Agricultural land, cultural woodlands and settlements make up the rest. The forest types are very diverse and range from dry sites with Scots pine to mesic sites with beech to wet sites with ash [*Fraxinus excelsior* L.] and black alder [*Alnus glutinosa* L.] [56]. Recently, a major part of this territory was designated as a MAB UNESCO Biosphere Reserve “Roztochya” [57, 58]. There are 120 settlements in Roztochya with about 60,000 inhabitants (Yavorivskiy and Zhovkivskiy rayons). The population density is about 80 persons per km^2^ [59]. The level of official unemployment for 2013 was 1.6 – 2 %, (yet actual rates might be much higher) which is a major problem in the region [60]. The healthcare system and its health centres are funded by the state and are theoretically free to all citizens. However, the cost of medicines and treatments often has to be covered by the patient. There are also some private health care centres in the area. However, their services are exceedingly expensive for many people in rural areas.

Kortkeros (60°45′ N - 62°50′ N and 50°45′ E - 53°30′ E) occupies 19,700 km^2^ (Fig. 1). The forest cover is approximately 90 % [61]. The boreal forest is formed by Scots pine and Norway spruce in later successional stages, but with a domination of birch [*Betula* spp.] and aspen [*Populus tremula* L.] in young and middle-aged forests. The human population density is low, less than one person per km^2^, and most of the population lives in rural areas. There are 60 villages with a total of 19,200 inhabitants [62]. The low population density in the area is both due to a low birth rate and a high emigration rate. A high level of unemployment (21 % officially) is a main problem [62]. Health care is free to all citizens but accessibility is low. The only regional hospital is located in the village of Kortkeros. Patients have to cover all costs for medicines themselves and the prices are relatively high.

### Interviews

In total 205 semi-structured interviews [63, 64] were conducted with local people in the three study areas (60 in Småland, 90 in Roztochya and 55 in Kortkeros) during a total of 6 months of field work from 2010 to 2013. Local inhabitants were selected for interviews using a convenience sample in the three study areas. Interviewees were chosen among people who were met in the forest near villages or on the village’s street. First, the study was introduced and oral consent was obtained, along with a permit to record the interview. National or ethnic belonging was not asked, but was recorded if the respondent provided that information voluntarily. The uneven number of respondents among the study areas was due to the fact that the diversity of answers among the respondents in Roztochya was higher in comparison with the Swedish and the Russian study areas, and a larger sample of respondents was required in order to reach the data saturation point when no new information was provided by additional interviews [65].

The semi-structured interview manual consisted of open-ended questions and specific questions for confirmation. Questions focused on (1) the species of plants and fungi that are harvested from the forest; (2) the amount harvested, (3) current personal uses and practices, and (4) changes of over time, (5) sources of knowledge regarding the use of those species as wild food and medicine, (6) traditional recipes of dishes and medicine from NWFPs. Using open-ended questions, interviewees had full freedom to talk about NWFPs from their own perspective. Respondents were asked to divide NWFPs that they used into wild food and medicine. This was asked because the line between wild food and medicine is not always easy to draw and NWFPs are often used both as medicine and food. Pieroni *et al.* [28] conceptualised a continuum, ranging from species that are only used for food, through multipurpose food and medicinal species, to exclusively medicinal species. In addition, species that are consumed without any unique specification of their assumed health benefit, because they are considered to be ‘healthy’ in general, are called ‘functional foods’ [30]. The project followed the ethical guidelines outlined by the American Anthropological Association [66] and the International Society of Ethnobiology [67].

All respondents were interviewed in their native language (Swedish, Ukrainian and Russian) by the first author. In Småland the interviews were conducted with the help of an interpreter. The interviews lasted between 15 and 110 min and were digitally recorded (Olympus DM-20), and then fully transcribed. The interview transcripts were analysed for emergent themes related to the research questions.

### Identifying plant species and uses

Plant species were identified during interviews in the field by the first author who has been botanically trained at university level. Interviews took place either in the forest or at the homes of peoples. If interviews took place inside and the local plant/fungi name was unknown to the researcher, additional information or local synonyms were asked for during the interviews. In Roztochya and Småland, respondents went outside during interviews and pointed out the discussed species, if needed. This was possible due to the seasonal timing of the interviews. In Kortkeros, however, the fieldwork was done in late autumn, therefore additional questions had to be asked to clarify the names of species, some respondents used books with pictures and botanical names of the plants to identify the plants. Hence, since it was possible to identify all species through visual identification in the field or by asking additional questions, no voucher specimens were collected. Latin names of plant species were further verified based on Flora Europea [68] and International Plant Name Index [67]. Names of mushrooms species were verified based on SPECIES 2000 database [69]. An inventory of NWFPs harvested and used as wild food and medicine was compiled, based on all species that were mentioned during the interviews. We included wild species, semi-domesticated and domesticated wild species, such as *Mentha* spp. and *Sambucus nigra* L. in the study [3].

## Results

### Wild food used at home

#### Plant species

Respondents in all three case studies used a wide range of plant species as food. Harvested NWFPs were used both fresh and processed. Wild fruits and mushrooms were considered to be good for diversifying everyday meals.

Generally, all respondents collected wild fruits, specifically berries. The amount of collected species differed between informants and study areas (Table 1). In both the Ukrainian and Russian study areas the amount of collected fruits per household was higher than in the Swedish study area. But in some cases in Småland, respondents collected large amounts of wild fruits for consumption during winter time, as well as to share with friends and relatives. In both Roztochya and Kortkeross the respondents stated that it was hard to collect fruits (including berries), and define quantities as the harvest was dependent on yearly yields. Fruits were consumed fresh (sometimes as juice) or prepared to preserve (as we will discuss further).

**Table 1:** The number and amount of collected fruits and fungi species in the study areas

	Småland, Sweden	Roztochya, Ukraine	Kortkeros, Russian Federation
Maximum number of collected berry species per person	8	14	12
Average number of collected berry species per person	3	8	6
Maximum number of collected fungi species per person	9	13	12
Average number of collected fungi species per person	3	6	6
Amount of collected fruits (average/max), litres family/year:			
*Vaccinium myrtillus* L.	2–5/50	10/35	20/65
*Vaccinium vitis-idaea* L.	2–5/50	2/5	12
*Rubus* spp.	2/5	10/15	20
*Rubus idaeus* L.	-	10/20	-
*Fragaria vesca* L.	-	1–2/50	1
*Vaccinium oxycoccos* L.	-	1/2	30
*Rubus chamaemorus* L.			10
Amount of collected mushrooms (average/max), kg family/year:	1/50	3/130	3/200
*Boletus edulis* Bull.	1/6	3/130	3/30
*Cantharellus cibarius* Fr.	1/50	1/5	5/45
*Lactarius resimus* Fr.	-	-	15/200

Besides fruits (berries), birch sap was another popular form of wild food in the Russian and the Ukrainian study areas. In both areas, respondents collected the sap of birch [*Betula pendula* Roth. and *B. pubescens* Ehrh.] (from 3 to 10 l per household) for personal needs, as “a healthy and tasty drink”.

In addition, in Roztochya during spring times young leaves from common nettle [*Urtica dioica* L.] and *Primula* spp. (both *Primula veris* and *P. elatior*) were also collected and used in salads and soups. Common elder flowers [*Sambucus nigra* L.] were used to make a sparkling drink. Both *Rumex spp.* and *Urtica dioica* were used in soups by respondents in Roztochya. *Rumex acetosa* are commonly cultivated and *Urtica dioica* grows in the gardens, therefore, respondents did not view those species as wild food.

#### Fungi

Fungi were popular wild food in all three areas. The majority of respondents in Småland and all respondents in Roztochya and Kortkeros collected mushrooms (see Tables 2, 3 and 4). In Småland the most popular harvested mushrooms were: chanterelle [*Cantharellus cibarius* Fr.] and funnel chanterelle [*Craterellus tubaeformis* (Fr.) Quél.]. In Roztochya the most popular mushrooms were *Boletus* spp.^1^ Respondents also liked to collect sheep’s head [Both *Polyporus umbellatus* (Pers.) Fr. and *Grifola frondosa* (Dicks.) Gray], which is a Red Book listed species in Ukraine [70]. The most popular species in Kortkeros was *Lactarius* spp.

**Table 2:** Use of mushrooms in Roztochya (Ukraine)

Family	Species	English name	Local names	Food use
Agaricaceae	*Macrolepiota* spp.	Parasol mushroom	Гриб-парасолька, парасолька, гриб-зонтик	Whole mushroom
Agaricaceae	*Agaricus campestris* L.	Field mushroom	Печериця, шампіньйон	Whole mushroom
Boletaceae	*Boletus edulis* Bull.	Penny bun	Білий гриб, боровик, білий, справжній гриб	Whole mushroom
Boletaceae	*Leccinum aurantiacum* (Bull.) Gray	Red-capped scaber stalk	Підосиковик, червоноголовець, червнонюх, підосичник	Whole mushroom
Boletaceae	*Leccinum scabrum* (Bull.) Gray	Scaber stalk	Підберезовик, козар, козарик, бабка	Whole mushroom
Boletaceae	*Boletus badius* (Fr.) Fr.	Bay bolete	Польський гриб	Whole mushroom
Boletaceae	*Boletus chrysenteron Bull.*	Red cracking bolete	Моховик, решітка	Whole mushroom
Cantharellaceae	*Cantharellus cibarius* Fr.	Chanterelle	Лисичка	whole mushroom
Meripilaceae	*Grifola frondosa* (Dicks.) Gray	Sheep’s head	Бараняча голова, баранячка, бараньоха	Whole mushroom
Polyporaceae	*Polyporus umbellatus* (Pers.) Fr.	Sheep’s head	Бараняча голова, баранячка, бараньоха	Whole mushroom
Morchellaceae	*Morchella esculenta* (L.) Pers.	Common morel	Зморшок, сморшок	Whole mushroom
Physalacriaceae	*Armillaria mellea* (Vahl) P. Kumm.	Honey fungus	Опеньок	Whole mushroom
Russulaceae	*Lactarius resimus* (Fr.) Fr.		Груздь	Whole mushroom
Russulaceae	*Russula* spp.	Russula	Сироїжки	Whole mushroom
Suillaceae	*Suillus luteus* (L.) Roussel	Slippery jack	Маслюк	Whole mushroom

**Table 3:** Use of mushrooms in Kortkeros (Russian Federation)

Family	Species	English name	Local names	Food use
Agaricaceae	*Macrolepiota* spp.	Parasol mushroom	Гриб-зонтик, зонтик	Whole mushroom
Boletaceae	*Boletus edulis* Bull.	Penny bun	Белый гриб, боровик	Whole mushroom
Boletaceae	*Leccinum aurantiacum* (Bull.) Gray	Red-capped scaber stalk	Подосиновик красный, подосиновик, красноголовик	Whole mushroom
Boletaceae	*Leccinum scabrum* (Bull.) Gray	Scaber stalk	Подберёзовик обыкновенный, подберёзовик, чёрный	Whole mushroom
Boletaceae	*Boletus chrysenteron Bull.*	Red cracking bolete	Моховик	Whole mushroom
Cantharellaceae	*Cantharellus cibarius* Fr.	Chanterelle	Лисичка обыкновенная, лисичка	Whole mushroom
Discinaceae	*Gyromitra esculenta* (Pers.) Fr.	False morels	Строчок	Whole mushroom
Morchellaceae	*Morchella esculenta* (L.) Pers.	Common morel	Сморчок	Whole mushroom
Physalacriaceae	*Armillaria mellea* (Vahl) P. Kumm.	Honey fungus	Опёнок, собачий гриб	Whole mushroom
Russulaceae	*Lactarius pubescens* Fr.	Downy milk cap	Волнушка белая, волнушка	Whole mushroom
Russulaceae	*Lactarius resimus* (Fr.) Fr.		Груздь настоящий, груздь	Whole mushroom
Russulaceae	*Lactarius torminosus* (Schaeff.) Gray	Woolly milkcap	Волнушка розовая	Whole mushroom
Russulaceae	*Russula* spp.	Russula	Cироежки	Whole mushroom
Suillaceae	*Suillus luteus* (L.) Roussel	Slippery jack	Маслёнок обыкновенный, маслята, маслёнок	Whole mushroom
Suillaceae	*Suillus bovinus* (L.) Roussel	Jersey cow mushroom	Козляк	Whole mushroom
Tricholomataceae	*Tricholoma equestre* (L.) P. Kumm.	Yellow knight	Зеленушка	Whole mushroom

**Table 4:** Use of mushrooms in Småland (Sweden)

Family	Species	English name	Local names	Food use
Agaricaceae	*Macrolepiota* spp.	Parasol mushroom	Fnasig fjällskivling	Whole mushroom
Boletaceae	*Boletus edulis* Bull.	Penny bun	Karljohan	Whole mushroom
Boletaceae	*Leccinum aurantiacum* (Bull.) Gray	Red-capped scaber stalk	Aspsopp	Whole mushroom
Boletaceae	*Leccinum scabrum* (Bull.) Gray	Scaber stalk	Brun aspsopp	Whole mushroom
Cantharellaceae	*Cantharellus cibarius* Fr.	Chanterelle	Kantarell	Whole mushroom
Cantharellaceae	*Craterellus tubaeformis* (Fr.) Quel	Yellowfoot	Trattkantarell	Whole mushroom

#### Preparing for winter time: preserves

In order to overcome the season-dependent supply of wild foods, people in all three areas made preserves for winter times, such as different jams, juices and marmalades from wild fruits or marinated mushrooms. However, the diversity and quantities of preserves were higher in the Russian and Ukrainian study areas than in the Swedish case study. In Småland, some respondents made preserves from mushrooms, bilberries and cowberries for personal consumption during winter times. Chanterelles were frozen and consumed during winter time and funnel chanterelle were used dried for stews and sauces in the Swedish study area. However, most respondents collected chanterelle and funnel chanterelle once or twice per year for immediate cooking. Cowberry jam was used as an accompaniment to meat dishes and pancakes in Småland.

In both the Ukrainian and Russian cases jam, marmalade and juice made of bilberries, wild strawberries and raspberries were popular among respondents. In Roztochya preserves were additions for the everyday menu. *Boletus* species were dried or marinated (pickled) and dishes containing this species were considered a delicacy. *Armillaria* spp. was mainly used marinated. *Russula* spp. was used only fresh for immediate cooking.

Furthermore, respondents shared recipes for homemade honey made of dandelion flowers [*Taraxacum officinale* Weber ex Wiggers], which is considered to be a very tasty jam. The Ukrainian respondents mentioned that due to the increased availability of good quality freezers, they preferred deep-freezing mushrooms over marinating as a preservation technique for winter times.

All respondents in the Russian study area salted and pickled *Lactarius* spp., while marinated *Boletus* spp. was popular as well. Lacto-fermented *Lactarius* spp. were reported as a delicacy and a part of rural cuisine for centuries. During recent years, due to the availability of freezers, frozen mushrooms were also widely used. Respondents pointed out that *Cantharellus cibarius* was not used by native Komi people in the past, and local consumption started recently. Russian respondents froze cowberries and cranberries to preserve them for winter times. The frozen fruits would ‘taste as if they were freshly picked’. The majority of respondents in the Russian study area prepared jam from cloudberries (*Rubus chamaemorus* L.), which they considered a delicacy. Dried fruits of *Prunus padus* were used for a traditional drink, called ‘lyazj’. Wild *Ribes nigrum* and R. *rubrum* were used for jam and marmalade. Traditional cookies called ‘shanechky’ or ‘shangy’ were made with jam made of bilberries or cowberries.

#### Past versus present

For Swedish respondents the use of NWFPs is not as important as it used to be for earlier generations. Interviewees stated that 20 years ago it was more common to pick different wild fruits and mushrooms for food. Respondents pointed out that, nowadays, one can buy everything from a store. Among Swedish respondents it is common to give collected fruits as a present to friends or family members. Contrarily, for Ukrainian and Russian respondents the use of NWFPs also played an important role for their livelihoods.

Additionally, respondents in the Ukrainian study area mentioned that it is still a tradition to cook dishes using NWFPs for religious holidays such as Christmas. For Christmas, respondents mentioned a traditional vegetarian dinner, including the special dish *varenyky* (or ravioli, *i.e.*, small squares of pasta containing a mixture of mushrooms, potatoes or cheese) with mushrooms (called *vushka*) served with beetroot soup (*borzch*), and potatoes with mushrooms sauce. This tradition is considered important even by respondents who no longer collected mushrooms as a subsistence activity. In Roztochya one respondent named *Chenopodium* spp., which was used as “famine food” during the times of World War II.

##### The forest as apothecary: wild species used as medicine

In Småland, local people no longer collected medicinal herbs for curative treatments. Respondents often mentioned that they did not have enough knowledge about medicinal plants and fungi. Instead they go to the forest for recreational activities to relief stress, and to get energized. Participants mentioned that they collected wild fruits because they were rich in vitamins. These functional food uses could be considered medicinal in a broad (preventive) sense. People in all study areas considered fruits to be rich in vitamins and thus good for one’s health. Respondents in the Ukrainian and Russian study areas collected fruits for their kids because they considered it a natural and healthy product. Thus, some species were only used as food (Tables 5 and 6), while other species were used both as wild food and medicine. This example of functional foods illustrates that it is often hard to distinguish between the use of NWFPs as wild food or medicine. Some species were exclusively used as medicine (such as *Arctium lappa* L., *Tussilago farfara* L., *Plantago major* L., *Chamomilla recutita* (L.) Rauschert, *Elymus repens* (L.) Gould).

**Table 5:** Use of medicinal herbs in the Roztochya (Ukraine)

Family	Species	English name	Local names	Part used	Mode of preparation	Medicinal use	Food use	Level of use
APOCYNACEAE	*Vinca minor* L.	Lesser periwinkle	Барвінок малий, барвінок	fl, ap	Tincture	Eye problems, blood cleaning, blood coagulation	-	+
ARACEAE	*Acorus calamus* L.	Calamus	Аїр, шавар	r	Tincture	Problems with gastrointestinal tract, gangrene	-	+
ARISTOLOCHIACEAE	*Asarum europaeum* L.	European wild ginger	Kопитняк	l, r	Tincture	Heart, bronchiae, nervous problems	-	+
BETULACEAE	*Betula* spp.	Birch	Береза	bd, sa	tea, tincture	Kidneys, cosmetic for hair, skin, vitamins	-	+
BORAGINACEAE	*Symphytym officinale* L.	Comfrey	Живокіст	ap	Liquids with alcohol, tincture	Arthritis (‘when hands hurt’)	-	+
CANNABACEAE	*Humulus lupulus* L.	Hops	Хміль	fr	Infusion, boiled tincture	Cosmetic use (hair conditioner), hair loss, blood pressure, liver disease	-	+
CAPRIFOLIACEAE	*Sambucus nigra* L.	Elderberry	Бузина чoрна	fl	Tincture	Cough	-	+
	*Viburnum opulus* L.	Guelder rose	Калина звичайна	b	Tincture	Blood pressure, vitamins, immunity improving, cough	Fruits	+
COMPOSITAE	*Achillea millefollium* L.	Yarrow	Деревій, тисячолистник	fl, ap	Tincture	Wound treatment, haemostatic, blood coagulation	-	+
	*Anthemis maritima* L.	Maritime chamomile	Романець	fl	Infusion	Anthelmintic	-	+
	*Arctium lappa* L.	Greater burdock	Лопух	r	Infusion, tincture	Cosmetics, for ‘nice shining’ hair and hair growth	-	+
	*Artemisia absinthium* L.	Wormwood	Полин, полин гіркий	ap	tincture	Stomach ailments, anthelmintic, fleas	-	+
	*Bidens tripartita* L.	Three-lobe beggarticks	Череда	l	Bath	Skin disease, for babies bath	-	+
	*Calendula officinalis* L.	Marigold	Календула, нагідки	fl	Infusion, tincture	Antiseptic, tonsillitis	-	+
	*Chamomilla recutita* (L.) Rauschert	Chamomile	Ромашка, рум’янок	fl, ap	Infusion, tincture	Stomach ailments,epatitis, cosmetics (skin softness), to treat burns	-	++
	*Centaurea cyanus* L.	Cornflower	Волошки сині	fl	Infusion	Urinary tract	-	+
	*Helichrysum arenarium* (L.) Moench	Dwarf everlast	Безсмертник, цмин	fl	Infusion	Antiseptic, tuberculosis, hepatitis	-	+
	*Taraxacum officinale* Weber ex Wiggers	Dandelion	Кульбаба,	fl	Liquids with alcohol, tincture	Stomach disease, cosmetics to treat black spots and freckles	Flowers	++
	*Tussilago farfara* L.	Coltsfoot	Мати й мачуха	fl, l	Tincture	Asthma, tuberculosis, stomach ailments, cosmetics for hair	-	+
CRUCIFERAE	*Capsella bursa-pastoris* (L.) Medik.	Shepard’s purse	Грицики звичайні	ap	Infusion	Blood coagulation	-	+
ERICACEAE	*Arctostaphylos uva-ursi* (L.) Spreng.	Bearberry	Медвежі вушка	l	Infusion	Kidneys, uterine bleeding, women’s diseases	-	+
	*Calluna vulgaris* (L.) Hull	Heather	Верес	ap	Honey	Asthma, rheumatism	-	+
	*Vaccinium myrtillus* L.	Bilberry	Чорниця	b, l	Infusion, food	Vitamins, diabetes, stomach	Fruits	+++
	*Vaccinium oxycoccos* L.	Cranberry	Клюква	b	Eaten fresh, jams	Vitamins	Fruits	+
	*Vaccinium vitis-idaea* (L.) Avror	Cowberry	Брусниця	b, l	Juice, infusion	Vitamins, kidneys problems	Fruits	+
GENTIANACEAE	*Centaurium erythraea* Rafn	Common centaury	Центорія	fl, ap	Infusion	Stomach ailments	-	+
GRAMINEAE	*Elymus* spp.	Couch grass	Пирій	l	Tincture	Urinary tract	-	+
GUTTIFERAE	*Hypericum perforatum* L.	St John’s worth	Звіробій	fl, ap	Tincture, infusion	Stomach ailments (when stomach hurts), inflammations	-	+++
HIPPOCASTANACEAE	*Aesculus hippocastanum* L.	Chestnut	Каштан кінський	fl	Tincture	Arthritis	-	+
LABIATAE	*Mentha* spp.	Mint	М’ята	ap	Tincture, infusion	Toothache, sedative, analgesic	-	++
	*Glechoma hederacea* L.	Ground-ivy	Розхідник	l	Infusion	Cold, caught, tuberculosis	-	+
	*Lamium album* L.	White nettle	Духмяна кропива	fl	tea	Uterine bleeding, after having baby	-	+
	*Origanum vulgare* L.	Oregano	Материнка	fl, ap	Tincture	Gynaecological disease (emmenagogue), breathing problems	-	+
	*Prunella vulgaris* L.	Common self-heal	Cуховершки	fl	Tincture	For throat rinsing	-	+
	*Thymus* spp.	Thyme	Чебрець, чабрець, цебрик	ap	Liquids with alcohol, tincture, infusion	Antiseptic, blood pressure reduction, anti-hypertension	-	++
LEMNACEAE	*Lemna minor* L.	Common duckweed	Ряска	ap	Infusion	Psoriasis, asthma	-	+
LILIACEAE	*Allium ursinum L.*	Bear’s garlic	Черемша	ap	Tincture, infusion	Antiseptic, stomach infections, rheumatism	-	+
	*Convallaria majalis* L.	Lily of the valley	Конвалія	fl	tinctures	Heart disease	-	+
LORANTHACEAE	*Viscum* spp.	Mistletoe	Омела	l	Tincture	Blood pressure, immunostimulant	-	+
LYCOPODIACEAE	*Huperzia selago* (L.) Bernh. ex Schrank & Mart.,	Northern firmoss	Баранець	ap	Tincture	Alcoholism treatment	-	+
ONAGRACEAE	*Epilobium angustifolium* L.	Fireweed	Іван Чай	l	Infusion	Headache, blood coagulation	-	+
PAPAVERACEAE	*Chelidonium majus* L.	Greater celandine	Чистотіл	ap	Tincture	Cancer treatment	-	+
PINACEAE	*Pinus sylvestris* L.	Pine	Сосна звичайна, сосна	bd	Tincture with alcohol	Cough, asthma, cold, vitamin C	-	+
PLANTAGINACEAE	*Plantago major* L.	Greater plantain	Подорожник	ap	Tincture	Stomach ailments, breathing problems (tuberculosis)	-	+
POLYGONACEAE	*Polygonum aviculare* L.	Сommon knotgrass	Cпориш звичайний	ap	Infusion	Urinary tract, gastritis, kidneys	-	+
PRIMULACEAE	*Primula veris* L.	Сommon cowslip	Первоцвіт	ap	Tincture, infusion	Cough	-	+
ROSACEAE	*Crataegus* spp.	Hawthorn	Глід	f,fl	Tincture	Heart, *i.e.*, hypertension or regulation of blood pressure, sedative	Fruits	+
	*Fragaria vesca* L.	Strawberry	Суниці лісові, суниці	b,l,f	Tincture	Flu and cough remedy, vitamins	Fruits	+++
	*Potentilla alba* L.	White cinquefoil	Перстач білий	r, ap	Tincture	Treatment of thyroid	-	+
	*Potentilla erecta* (L.) Hampe	Common tormentil	Перстач прямостоячий	r	Tincture	Gastritis, peptic ulcer	-	+
	*Potentilla reptans L.*	Creeping cinquefoil	Перстач повзучий	ap	Tincture	Blood coagulation	-	+
	*Prunus padus* L.	Hackberry	Черемха звичайна, черемха	f,ba	Infusion, tincture	Astringent	Fruits	+
	*Prunus spinosa* L.	blackthorn	Терен	f	Liquids with alcohol, tincture	Stomach disease, depurative	Fruits	+
	*Pyrus communis* L.	European pear	Лісова грушка	br,fl	Infusion	Treatment of joints	Fruits	+
	*Rubus ideaus* L.	Raspberry	Малина	l,b	Fresh, frozen, tea, tincture	Flu and cough remedy, high temperature	Fruits	+++
	*Rosa* spp.	Dog rose	Шипшина	fl		Vitamins, immunity improving (immunostimulant) tea	Fruits	++
	*Rubus* spp.	Wild blackberries	Види ожин, ожина	b	Juice, infusion (f)	Vitamins, food, wine producing	Fruits	+++
	*Sanguisorba officinalis* L.	Great burnet	Родовик	r	Tincture	Blood cleaning blood coagulation	-	+
	*Sorbus aucuparia* L.	Rowan	Горобина звичайна, горобина	b	Juice, tincture	Blood pressure normalization	Fruits	+
THYMELAEACEAE	*Daphne mezereum* L.	Mezereon	Вовчі ягоди, вовче лико	ba	Poisonous, use externally	Arthritis, cold	-	+
TILIACEAE	*Tilia* spp.	Linden	Липа	fl	Infusion	Vitamins, flu, antipyretic	-	++
UMBELLIFERAE	*Levisticum officinale* W.D.J.Koch	Lovage	Любисток	ap	Tincture	Cosmetics (herb for bath), skin, stomach, antiseptic,Liver, kidneys Cosmetics	-	+
URTICACEAE	*Urtica dioica* L.	Stinging nettle	Кропива дводомна, кропива жалка, кропива	l	Infusion, tincture	Vitamins, cosmetic, blood coagulation, mouth cleaning (mouth wash/ oral hygiene)	Leaves	+

**Table 6:** Use of medicinal herbs in the Kortkeros (Russia)

Family	Species	English name	Local names	Part used	Mode of preparation	Medicinal use	Food	Level of use
BETULACEAE	*Betula* spp.	Birch	Берёза	bd, sa	Liquids with alcohol, tincture	Panacea (‘100 diseases’) *e.g.*, small scratches, flu	-	++
CAPRIFOLIACEAE	*Viburnum opulus* L.	Guelder rose	Калина обыкновенная калина	b	Infusion, tincture	Vitamins, cough	Fruits	+
COMPOSITAE	*Achillea millefollium* L.	Yarrow	Тысячелистник обыкновенный	l, fl	Infusion	Blood coagulation	-	+
	*Arctium lappa* L.	Greater burdock	Лопух	r	Decoction	For strengthening hair (conditioner)	-	+
	*Artemisia absinthium* L.	Wormwood	Полынь	l, fl	Tea	Stomach ailments	-	+
	*Chamomilla recutita* (L.) Rauschert	Chamomile	Ромашка аптечная, ромашка	l, fl	Infusion	Good for health	-	+
	*Taraxacum officinale* Weber ex Wiggers	Dandelion	Одуванчик лекарственный, одуванчик	l, fl	Infusion, tincture	Vitamins, headache	-	+
	*Tussilago farfara* L.	Coltsfoot	Мать-и-мачеха	fl, l	Infusion	Cough	-	+
	*Tanacetum vulgare* L.	Tansy	Пижмо	l, fl	Tincture	Stomach ailments, diarrhea	-	+
CRASSULACEAE	*Rhodiola rosea* L.	Golden root	Золотий корінь	r	Tincture (d)	General ‘for good health’	-	+
CUPRESSACEAE	*Juniperus communis* L.	Common juniper	Можжевельник	r	Tincture	For good health	-	+
EQUISETACEAE	*Equisetum* spp.	Horstail	Хвощ	l	Infusion	Kidneys and urinary tract	-	+
ERICACEAE	*Ledum palustre* L.	Wild rosemary	Трава богульник	ap	Tincture (must be stored for 3 years, otherwise poisonous)	Asthma	-	+
	*Vaccinium myrtillus* L.	Bilberry	Черника	b	Tincture	Vitamins, eyes disease, problems with stomach	Fruits	+++
	*Vaccinium oxycoccos* L.	Cranberry	Клюква	b	Eaten fresh, jams, frozen	High blood pressure, vitamins	Fruits	+++
	*Vaccinium vitis-idaea* (L.) Avror	Cowberry	Брусника	b, l	Infusion	Vitamins, flu, kidneys, diuretics	Fruits	+++
GRAMINEAE	*Elymus* spp.	Couch grass	Пырей	r	Tincture	Kidneys, breathing problems, reproductive system	-	+
GROSSULARIACEAE	*Ribes* spp.	Blackcurrant, redcurrant	Смородина	b, l	F: infusion, juice	Rich in vitamins	-	++
GUTTIFERAE	*Hypericum perforatum* L.	St John’s worth	Зверобой	l (d)	Tea, tinctures	Problems with stomach	-	+
LABIATAE	*Mentha* spp.	Mint	Мята	l, fl	Tincture, infusion	Nervousness, stomach	Leaves	+
	*Thymus* spp.	Thyme	Тимьян	l, fl	Infusion (f, d)	For strong health, immunostimulant	-	+
LILIACEAE	*Polygonatum odoratum* (Mill.) Druce	Angular Solomon’s seal	Купина	l, fl	Tincture	Rheumatism, diabetes	-	+
MENYANTHACEAE	*Menyanthes trifoliata* L.	Bog-bean	Трилисник	l	Tea	Stomach, gastritis	-	+
ONAGRACEAE	*Epilobium angustifolium* L.	Fireweed	Иван-чай	l	Infusion	Cancer treatment	-	+
ORCHIDACEAE	*Cypripedium calceolus* L.	Lady’s slipper orchids	Венерин башмачок	r	Tea	Headache	-	+
PAEONIACEAE	*Paeonia anomala* L.	Paeonia	Сибірський піон	r	Tincture	For good health	-	+
PINACEAE	*Abies alba* Mill.	Silver fir	Пихта	bd	Brooms for sauna, steam bath	Immunostimulant	-	+
PLANTAGINACEAE	*Plantago major* L.	Greater plantain	Подорожник	l	Tincture	Small scratches	-	+
ROSACEAE	*Comarum palustre* L.	Purple marshlocks	Сабельник	l, fl	Tincture	Arthritis, rheumatism, cancer	-	+
	*Filipendula vulgaris* Moench	Dropwort	Таволга	fl, l	Infusion, tincture	Antiseptic, arthitis, heart, skin disease	-	+
	*Prunus padus* L.	Hackberry	Черёмуха обыкновенная, черёмухa	fr	Dried fruits, infusion from dried fruits	Pain in stomach, diarrhoea	Fruits	++
	*Rubus ideaus* L.	Raspberry	Малина	br, fr, l	Tincture	Vitamins, flu, high temperature	Fruits	++
	*Rosa* spp.	Dog rose	Шиповник	fr	Infusion	Flu, vitamins, rich in vitamin C, for immunity	Fruits	+++
	*Rubus chamaemorus* L.	Cloudberry	Морошка	b, fl se	Infusion	Vitamins, flu, cough	Fruits	+++
	*Sorbus aucuparia* L.	Rowan	Рябина обыкновенная, рябина	fr	Juice, dried fruits	High blood pressure, stomach problems	Fruits	+++
SALICACEAE	*Salix* spp.	Willow	Ива	ba	Infusion	Natural aspirin, rheumatism, ostheochondrosis	-	+
SCROPHULARIACEAE	*Euphrasia officinalis* L.	Eyebright	Очанка	r	Infusion (d)	For eyes disease	-	+

*ap* aerial parts (herb), *b* berries, *ba* bark, *bd* buds, *br* branches, *fl* flowers, *fr* fruit, *l* leaves, *r* root, *sa* sap, *se* sepals, *f* fresh, *d* dried

- No use, + used by up to 10 respondents, ++ 10–20 respondents, +++ more than 20 respondents. Listed illnesses refer to emic categories

Unlike in Sweden, in the Ukrainian and Russian study areas, the collection of medicinal herbs was as popular as collecting wild food. The medicinal herbs used in Roztochya and Kortkeros are presented in Tables 5 and 6. The most popular species were raspberries [*Rubus idaeus* L.], guelder rose [*Viburnum opulus* L.], common hawthorn [*Crataegus* spp.] and rowan [*Sorbus aucuparia* L.], wild strawberries [Fragaria vesca L.], common nettle and dog rose [*Rosa canina* L.]. Different plant parts were used, such as the flowers of linden [*Tilia cordata* Mill.], the buds of birch tree [*Betula pendula* Roth.] and the leaves of common nettle.

In the Russian study area, the most used medicinal herbs were: cloudberries, cowberries, bilberries, raspberries, St John’s wort [*Hypericum perforatum* L.] and greater plantain [*Plantago major* L.]. The Russian respondents considered it important to use medicinal herbs during winter times to prevent flu and common colds.

Respondents in both Ukraine and Russia used medicinal herbs either as infusions or they prepared different kinds of tinctures for ‘promoting health’. Most medicinal plants were collected, dried in a dark place, and used for making an infusion. As a rule, dried herbs had to be infused for one hour before drinking. For preparing a tincture, additional ingredients were used, such as alcohol, sugar or honey. In addition, some medicinal treatments from animal origin were used in Kortkeross. Fat from the brown bear [*Ursus arctos*, Linnaeus, 1758] was used as a treatment for many different ailments. Fat from the European badger [*Meles meles*, Linnaeus, 1758] and marmot [*Marmot* spp.] were known to be used to treat tuberculosis.

In general, respondents in the Ukrainian and Russian study areas preferred to use medicinal herbs against certain illnesses rather than allopathic medicine from the pharmacy or shops. They also considered herbal remedies to be more environmentally friendly than pharmaceuticals. Moreover, interviewees often stated that their income was low and accordingly, pharmaceuticals were considered very expensive, while the cost associated with collecting medicinal herbs was much lower. Interestingly respondents reported their active use of medicinal herbs had increased since the collapse of the Soviet Union in 1991. Ukrainian respondents also mentioned cultural traditions and knowledge as important reasons for collecting wild food.

The most common ailments cured with herbal remedies in the Ukrainian case were flu, cough and gastrointestinal problems (Table 5). In addition, herbal remedies were used as vitamins, immuno-stimulants and cosmetics. Medicinal herbs were also used to treat chronic diseases like diabetes and hypertension (‘high blood pressure’) (Table 5). The most common ailments cured with herbal remedies in the Russian study area were rheumatism and arthritis, upper respiratory tract infections (cough and common cold), kidney and urinary tract problems, high blood pressure, blood coagulation problems and different gastrointestinal problems (stomach ailments, inflammation, gastritis) (Table 6). Remarkably, several respondents in the Russian and Ukrainian study areas claimed that a tincture of *Amanita muscaria* contains anti-carcinogenic properties.

### Wild food species collected for commercial purposes

Selling wild food was a widespread activity in Roztochya and Kortkeros; in Småland on the other hand, people only harvested wild food species for personal use. Many Swedish respondents stated that the sale of wildly collected fruits and mushrooms was important for rural livelihoods in the Småland region about 60–70 years ago.

The majority of respondents in the Ukrainian and Russian study areas mentioned that the collection of edible NWFPs for sale has become more intensive compared to about 20–25 years ago, prior to the collapse of the Soviet Union. During the Soviet period, people had a job at the collective farms, forestry or in the industry and there was neither time nor a need to collect NWFPs to get an additional income. However, collective farms and many industries were closed during the 1990s after the collapse of the Soviet Union. At the time of this study, unemployment in both regions was high. The forest provided an interesting opportunity to support local livelihoods. Accordingly, the majority of interviewees in the Ukrainian and Russian study areas collected fruits and mushrooms for selling. In Roztochya people sold NWFPs on local markets to consumers mainly from urban areas. The most frequently collected fruits for sale were: wild strawberries, bilberries, blackberries and raspberries. The most popular mushrooms collected for sale were the penny bun or cep [*Boletus edulis* Bull.], red-capped scaber stalk [*Leccinum aurantiacum* (Bull. ex St. Amans)] and honey fungus [*Armillaria* spp.]. In Roztochya, the distance to markets varied from two to 60 km. In villages located close to the border with Poland, locals often sold wild fruits (mostly bilberries) to Polish companies, who then transported the fruits to Poland for the production of value-added products. According to Stryamets *et al.* [18], villagers earn about the equivalent of two monthly salaries in rural areas per season from selling wild fruits (approximately 300 EUR). By picking and selling fruits, respondents could easily earn more than the average daily labour payment in rural areas (approximately 10 EUR).

In Kortkeros local people sold harvested NWFPs mainly to companies that freeze fruits and mushrooms for further transport. Additionally, as the nearest town with a market was up to 60–120 km away, in each village there were places where people sold their NWFPs to each other and to rare tourists. The respondents mainly sold fruits (bilberries, cowberries, cloudberries and cranberries). Locally collected penny bun and chanterelle were mainly sold at collecting stations to company representatives. The amount of fruits that were sold during the season in Kortkeros varied a lot, depending on the annual yield and on a collector’s employment status. Respondents pointed out that they generally collected as much as possible. Several respondents stated that they used their vacation time to pick wild fruits and mushrooms for selling. The minimum sold amount of fruits reported was 100 kg of cowberries. The maximum was 6 t of bilberries and cowberries (sold by one family in one season) and 1,5 t of mushrooms. Some people earned up to 250 000 rubles (approx. 5336 EUR) per season (the maximum sum that was mentioned in 2013).

## Discussion

### Wild food and medicine for recreation and economic survival

In all three study areas, interviewees highly appreciated wild food. However, our results indicate that the role of NWFPs as a source of wild food and medicine has a different importance for rural residents in the studied cases.

In Sweden, the current use of wild food and medicine mainly serves recreational purposes. However, historically NWFPs - except for mushrooms - were extensively used as wild food and medicine for centuries [71]. The custom of eating mushrooms was imported from France and adopted by the Swedish nobility in the 18^th^ century. Peasants began using mushrooms as a free food resource only after World War I and many species were used also for medicine [15, 19]. Our Swedish case study reflects a general trend in Western Europe, where there is a growing interest and demand for organic and natural products provided by forests [3, 72]. In Sweden there is a growing market for medicinal herbs in drug stores as there has been a strong upswing in the dietary supplement market. Nevertheless as other studies also indicated rural people do not collect NWFPs for commercial purposes, and most value-added products from NWFPs sold in Sweden, are produced in other countries [73].

In Sweden, the process of urbanization is one of the fastest in Europe [74]. This rapid urbanization is one of the reasons that many people have become disconnected from nature, which has been proven to cause more stress [75, 76]. Diseases caused by stress and physical inactivity are currently one of the main reasons of illness in the developed world [76–78]. At the same time, many scientific studies have shown that interactions with nature can provide a positive influence on mental and physical health and self-awareness [77, 79–81]. Moreover, multiple studies have indicated that activities undertaken in forests reduce stress [82–84] and the amount of the stress hormone cortisol [85]. Wild food collection and consumption provide healthier food, physical activity and stress relief. Most respondents in the Swedish study area indeed stated that collecting wild food has a very positive effect on their mental health. It helps them to reduce stress and provides a break from their daily routine, as collecting NWFPs is accompanied by physical activity, fresh air, relaxation and a general enjoyment of nature. Thus, these forest-based activities provide preventive, treatment and therapeutic health benefits [86, 87].

By contrast, the Ukrainian and Russian case studies illustrate how wild food and medicine can be important for personal food security and as an additional - and in many cases, the only - financial income. Due to political, social and economic developmental challenges in countries transitioning from planned to market economies in Eastern Europe, forest functions, other than wood production, have remained or regained great local and regional importance in the study areas. In Ukraine and NW Russia the use of NWFPs as wild food and traditional medicine has been important for centuries, and for the larger part of the 20^th^ century [35, 48, 88, 89]. NWFPs were particularly important during famines in the 19^th^ and 20^th^ centuries in Ukraine [6, 35] and because of food scarcity during the 20^th^ century in the Republic of Komi [48, 49]. Currently rural residents in both Roztochya and Kortkeros use NWFPs to supplement their diets and household income, notably during certain seasons of the year, and to help meet medical treatment needs. These resources are important for subsistence and as an additional income during hard times of economic transition, especially considering the high level of unemployment in these rural areas. Thus, NWFPs were important to avoid poverty in the Russian and Ukrainian rural study areas, which has also been shown to be the case in other parts of the world [42, 90–93]. By offering supplemental income (both subsistent and economic), collecting wild food and medicine provides a safety net or a risk management activity during economic crises [46, 90, 94, 95]. According to Chukwuone and Okeke [96] NWFPs contribute to food security in two ways (1) through direct consumption of NWFPs for local diets and (2) through trade of NWFPs to generate income. This view was corroborated by interviews in the Russian study area, which indicate that people survived during economic crisis in the 1990s using NWFPs. Currently, rural households’ dependence on wild fruits and mushrooms in Russia is much higher than in Sweden and Ukraine, which is also shown by other studies [72, 97].

The use of wild food and medicine may be related to the socio-economic situation in a country. In a country like Sweden with high living standards and a modern and freely available health care system, the collection, processing and consumption of wild medicine among respondents is limited and has a mainly recreational character. On the other hand, in countries of Eastern Europe such as the Ukraine and Russia which are in transition from planned to market economy with political and economic crises, financial insecurity and expensive medical care, the use of both wild food and medicine is still very active [13, 18]. In fact the local livelihood strategies of rural forest dependent communities are often based on the use of NWFPs.

### Traditional knowledge of wild food and medicine: lost in time?

Despite the importance for recreation or subsistence, in all three study areas traditional knowledge related to NWFPs was reported to be decreasing, due to shifts in lifestyles and interests. Nearly 90 % of the respondents said that their parents had taught them to pick fruits and mushrooms. Most respondents indicated that nowadays the younger generations appear to have lost interest in forest activities such as collecting foods and medicines. In all three case studies mainly middle-aged and elderly respondents were interested in harvesting and collecting NWFPs, especially if the practice was a tradition in their families and they had lived permanently in the countryside. In the Ukrainian and the Russian study areas, knowledge about medicinal plants was transmitted from generation to generation. The use of medicinal herbs was more popular among the elderly, while the younger generation seemed to be losing interest in using these NWFPs in both study areas. In Ukraine there is a high interest in wild medicinal herbs, for example observed in several TV and radio programs that describe herbs and their uses. The respondents also mentioned so-called ‘green pharmacies’, pharmacies that only sell medicinal herbs (dried herbs, tinctures and mixes) and provide free consultations on how to use traditional medicines.

Our results are consistent with findings on changes in patterns of wild food and medicine use in other parts of the world [3, 42, 89, 96] that are associated with lifestyle changes, urbanization, large-scale farming, and less contact with nature [17, 37]. These rapid changes are a threat to traditional practices, and stress the need to document the traditional knowledge that still exists in rural areas of Eastern Europe [98, 99].

Our case studies in Ukraine and Russia indicate that traditional knowledge on preserving wild fruits and mushrooms for winter times are still actively maintained. Such traditional methods of winter preserves stem from the past when this was a useful tradition to ensure food security in harsh winter times [3, 13, 36, 37, 100], as these techniques of drying, freezing, marinating and making jams all improve shelf-life considerably [101]. Finally, our findings also suggest that traditional knowledge has a tendency to ‘survive’ much easier where it has an important role in subsistence such as in Ukraine and Russia (self-sufficiency), as opposed to Sweden [102].

## Conclusions

Our results indicate that the collection of wild food and medicine depends to a large extent on the socio-economic situation of the collector. In economically less developed rural areas like Roztochya and Kortkeros, collecting wild food and medicine continues to be an important part of local livelihoods. In these areas, NWFPs help ensure food security both directly and by providing an additional source of income. In these case studies the low quality and high costs of biomedical health care were brought up as main reasons for an increase in the consumption of medicinal herbs. In an economically more developed region such as Småland, collecting wild food is mainly a recreational activity with important stress-reducing health benefits. The promotion of wild food and medicine can be a ‘bridge’ between nature and people. The commercial and health benefitting potential of wild food and medicine could be considerably enhanced by drawing upon traditional knowledge and building on the sustainable system of use that local people often have created. Thus, NWFPs and their value-added processing have potential to support different dimensions of rural livelihoods in Europe.

## Footnotes

1 In Småland participants generally provided the species name, while in Roztochya and Kortkeros respondents just reported the genus but not always the species of the collected mushrooms. Thus, the total number of collected species might be higher. For instance *Russula* spp. includes more than 17 species growing in the Roztochya area, but was reported as one genus.
